# Identification of Major Sequence Types among Multidrug-Resistant *Staphylococcus epidermidis* Strains Isolated from Infected Eyes and Healthy Conjunctiva

**DOI:** 10.3389/fmicb.2017.01430

**Published:** 2017-08-04

**Authors:** Smrutiti Jena, Sasmita Panda, Kinshuk C. Nayak, Durg V. Singh

**Affiliations:** ^1^Infectious Disease Biology, Institute of Life Sciences Bhubaneswar, India; ^2^Bioinformatics Center, Institute of Life Sciences Bhubaneswar, India

**Keywords:** multidrug-resistant, *Staphylococcus epidermidis*, ACME, SCC*mec* typing, PFGE analysis, MLST-genotyping, diversity

## Abstract

We examined the presence of virulence and antibiotic resistance genes, *SCCmec* types and determined the genomic diversity among ocular *S. epidermidis* isolates (patients-23, healthy controls-29). PCR determined the presence of antibiotic resistance genes, virulence genes and *SCCmec* types among all isolates. MLST and PFGE determined the genomic relatedness among them. All isolates of *S. epidermidis* showed resistance to at least one class of antibiotics of which 48 isolates were multidrug resistant and carried ARGs. Thirty-five isolates were methicillin resistant and carried *mecA* gene. Majority of the isolates were resistant to fluoroquinolones and showed mutation in *gyrA, parC*, and *parE* genes, however, few isolates showed additional novel mutations in *parC* gene. Of the MRSE strains, 17 strains carried SCC*mec* type IV, four type V, two type II, and two UT4. Seven strains carried novel combination of *ccr* complex and SCC*mercury* element, not reported earlier. All the *S. epidermidis* strains harbored *icaA* and *icaD* genes, 47 carried ACME operon, and 50 contained *IS256*. A noteworthy finding was the presence of ST179 among 43% of infected eye isolates an observation rarely reported among *S. epidermidis*. PFGE and MLST analysis showed genomic diversity among them. Statistical analysis suggests that few healthy conjunctiva isolates had characteristics similar to infected eye isolates. *S. epidermidis* strains carrying *mecA* gene are multidrug resistant, virulent and diverse irrespective of sources of isolation. *IS256* cannot be used as marker to differentiate isolates of infected eye from healthy conjunctiva.

## Introduction

*Staphylococcus epidermidis* an opportunistic pathogen and member of coagulase negative Staphylococci (CoNS) are normal inhabitant of human skin, mucosal and ocular surfaces and cause hospital acquired infections (Graham et al., 2007; Rogers et al., 2009; Le et al., 2014). This organism can cause number of ocular diseases like bacterial endophthalmitis conjunctivitis, blepharitis, and keratitis (Melo et al., 2011; Schimel et al., 2013; Bispo et al., 2014; Park et al., 2015). The predisposing risk factors associated with infections are mostly use of contact lenses, ocular surgery, and ocular inflammatory diseases (Bourcier et al., 2003; Keay et al., 2006; Park et al., 2015). Although methicillin is not used in the treatment of eye infections, increasing resistance to methicillin is known to contribute significantly to the spread and persistence of multidrug-resistant strains in a given setting (Alekshun and Levy, 2007; Asbell et al., 2008; Cavuoto et al., 2008; Lichtinger et al., 2012). The alteration in penicillin-binding protein (PBP2a) confers resistance to methicillin (Hartman and Tomasz, 1984). The *mecA* gene encodes for PBP2a and is present on the mobile genetic element termed as staphylococcal cassette chromosome *mec* (SCC*mec*) that carries a set of recombinase genes (*ccr*) (Katayama et al., 2000). A combination of class of *mec* gene complex and the *ccr* gene complex types determines the SCC*mec* types and diversity among staphylococci (Kondo et al., 2007; Bloemendaal et al., 2010; Martínez-Meléndez et al., 2016). Methicillin-resistant *S. epidermidis* (MRSE) carrying untypeable SCC*mec* element are often reported (Ruppé et al., 2009; McManus et al., 2015).

Resistance to fluoroquinolones occurs due to mutation(s) in the quinolone resistance determining region (QRDR) of *gyrA, gyrB, parC* and *parE* (Yamada et al., 2008). There are three accomplished mechanisms of macrolide resistance. (i) Modification of the target site, (ii) efflux of antibiotics, and (iii) drug inactivation. Plasmid-borne drug-resistance genes encoding for ABC transporter superfamily efflux pump and *msrA* can mediate the export of macrolide (Leclercq, 2002). Other plasmid located genes, *tetK* confers resistance to tetracycline and *pC194, pC221*, and *pC223* inactivates chloramphenicol by converting it to 3- acetyl and 1,3-diacetyl derivatives (Trieu-Cuot et al., 1993; Trzcinski et al., 2000).

Pathogenicity of *S. epidermidis* is attributed to its ability to form biofilm comprised of polysaccharide, protein and eDNA, which confers resistance to antibiotics. During biofilm formation bacteria first, adhere onto abiotic polymeric substances mediated by polysaccharide intercellular adhesin (PIA) encoded by *icaADBC* locus (Schommer et al., 2011). Arginine catabolic mobile element (ACME) also play a significant role in the pathogenicity by enhancing the fitness of the bacteria by evading the host immune system (Otto, 2009).

Several molecular typing methods have been used to study epidemiology and clonal relationships of *S. epidermidis*; however, not a single technique alone could discriminate the bacteria because of differences in the degree of typeability, reproducibility, and discriminatory power (Tenover et al., 1994). Pulsed-Field Gel Electrophoresis (PFGE) is known as a “gold standard” and widely used for studying the epidemiological investigations (Hookey et al., 1998). Multi-Locus Sequence Typing (MLST) is currently used for long-term evolutionary research and to study population structure of *S. epidermidis* isolates (Bloemendaal et al., 2010; Martínez-Meléndez et al., 2016). Although *S. epidermidis* is rated as one of the emerging ocular pathogens and showing resistance to antibiotics; not much information is available on their molecular diversity. It is not clear how the isolates from healthy conjunctiva are related to and cause ocular infection.

We analyzed antibiotic resistance and virulence profiles of *S. epidermidis* isolated from infected eye and healthy conjunctiva during 2007–2011 at L.V. Prasad Eye Institute, Bhubaneswar, India. We used PFGE and MLST to determine the genomic diversity and correlated results with antibiotic resistance, SCC*mec* type, and virulence genes, respectively. Genetic and phenotypic data was used to determine whether isolates from healthy conjunctiva had characteristics similar to infected eye isolates.

## Materials and methods

### Ethical statement

The study was approved by Institutional Review Board (IRB) of LV Prasad Eye Institute (LEC/08/110/2009), and the data were analyzed anonymously and reported.

### Bacterial strains

Twenty-three non-duplicate *S. epidermidis* were isolated from patients with a variety of ocular infections at L.V. Prasad Eye Institute, Bhubaneswar, India, during 2007–2011. These isolates were from patients with keratitis (*n* = 13), endophthalmitis (*n* = 2), conjunctivitis (*n* = 2), marsupialization of cyst (*n* = 1), chronic dacryocystitis (*n* = 1), traumatic cataract (*n* = 1), canaliculitis (*n* = 1), blepharitis (*n* = 1) and graft infiltrate (*n* = 1). Also, 29 strains were isolated from asymptomatic healthy conjunctiva. The study was conducted following the guidelines mentioned in the Declaration of Helsinki. All the 52 isolates were identified by using biochemical tests including Gram staining, catalase production, fermentation of glucose and mannitol and ID32 STAPH strips using ATB™ NEW v.1.0.0 software on an ATB™ reader (bioMerieux, France). The amplification of *S. epidermidis nuc* gene confirmed the identity of isolates (Hirotaki et al., 2011). *S. epidermidis* strains ATCC 35984 and ATCC 12228 obtained from American Type Culture Collection (ATCC, Manassas, VA), were used as controls in this study. *Staphylococcus aureus* ATCC 25293 and *Pseudomonas aeruginosa* ATCC 27853 was used as quality control strains for antibiotic susceptibility testing.

### Antibiotic resistance and genetic characteristics

Antibiotic susceptibility was performed with *S. epidermidis* for 13 antibiotics; oxacillin, penicillin, chloramphenicol, ciprofloxacin, ofloxacin, gatifloxacin, moxifloxacin, erythromycin, clindamycin, gentamicin, tetracycline, vancomycin, and cefazolin by microbroth dilution methods according to the Clinical Laboratory Standard Institute (CLSI) guidelines (Clinical and Laboratory Standards Institute, 2015). The interpretation criteria for each antibiotic tested were those published previously as CLSI document M100-S25. Isolates showing resistance to three or more than three class of antibiotics were referred here as multidrug resistant (MDR).

Using published methods, PCR determined the presence of *blaZ, catpC221, catpC223, ermC, msrA, mphC, aac6*′*-aph2*′, *aph3*′, and *tetK* genes encoding for penicillin, chloramphenicol, erythromycin/clindamycin, gentamicin and tetracycline in both phenotypic-resistant and -susceptible strains of *S. epidermidis* (Schmitz et al., 1999; Schlegelova et al., 2008; Argudin et al., 2011; Duran et al., 2012). We determined the mutations in *gyrA, gyrB, parC*, and *parE* genes by sequencing the PCR product (Yamada et al., 2008).

PCR tested all isolates for the presence of *icaA, icaD, arcA*, and *opp3AB* genes to assess the presence of *ica* and ACME operons (Arciola et al., 2001; Diep et al., 2008). Each strain was given a particular ACME type depending on the presence of either of the genes (Hellmark et al., 2013). Also PCR determined the presence of insertion sequence element *IS256* (Gu et al., 2005).

### PFGE, SCC*mec* typing, and MLST

PFGE of *S. epidermidis* genomic DNA digested with *Sma*I was carried out by the protocol described for *S. aureus* by Centre for Disease Control and Prevention, Atlanta, USA. Banding patterns were determined by using Bionumerics software, version 7.1 (Applied Maths, Belgium) using the Dice index and un-weighted pair group method with arithmetic average (UPGMA) with 0.5% optimization and 1.5% position tolerance. Isolates showing similarity coefficient of up to 80% were considered belonging to similar pulsotype (Van Belkum et al., 2007).

Two Multiplex PCRs (MPCR-1 and MPCR-2) were performed by the method described previously using specific primers and assigned SCC*mec* type based on the combination of *mec* and *ccr* complexes (Kondo et al., 2007; Zong et al., 2011). Also, PCR determined the presence of SCC*mercury* element (Kondo et al., 2007).

Internal fragments of seven housekeeping genes, *arcC, aroE, gtr, mutS, pyr, tpi*, and *yqil* were amplified using specific primers (Thomas et al., 2007). Amplified fragments were purified using ExoSAP-IT (Affymetrix) and performed sequencing using Life Technology ABI sequencer model 3537 at the sequencing facility of Institute of Life Sciences, Bhubaneswar, India. Sequence alignments were done using MEGA 6. The sequences of the housekeeping genes were analyzed using Bionumerics software version 7.1, and a particular sequence type (ST) was assigned based on the nucleotide polymorphism. Minimum Spanning Tree (MST) was constructed using “MST for categorical data” as analysis template and accordingly assigned ST. We performed partitioning analysis according to the eBURST algorithm. Clonal complex (CC) analysis was performed according to the classification described by Miragaia et al. (2007).

### Indexing diversity

Simpson's index of diversity (SID) was calculated for all the three typing techniques (PFGE, MLST, SCC*mec* typing) as described by Simpson (1949).

### Statistical analysis

We performed Principal Coordinates Analysis (PCoA) and Discriminant Analysis (DA) using PAST program v2.17 (Hammer et al., 2001). We carried out the discriminant analysis using default values to confirm the hypothesis whether two groups of isolates are different. All the genotypic and phenotypic data of the strains are given in the supplementary material (Tables S1, S2).

## Results

### Antibiotic resistance pattern

Of the 52 strains, 35 strains were resistant to oxacillin and carried *mecA* gene (Table 1). However, all isolates were resistant to one or more than one class of antibiotics and showed 32 resistance patterns (Table S3). None of the isolates were resistant to vancomycin or cefazolin. Forty-eight strains were multidrug resistant (Table 1) comprising all isolates of MRSE from both sources, all MSSE from the infected eye and six from healthy conjunctiva (Table S3). Twenty-one isolates from the infected eye and 13 from healthy conjunctiva showed resistance to fluoroquinolones. However, two from healthy conjunctiva showed intermediate resistance to ofloxacin and ciprofloxacin (Table 2).

**Table 1:** Minimum inhibitory concentration and presence of antibiotic resistance genes of methicillin resistant and susceptible *S. epidermidis* isolated from the infected eye and healthy conjunctiva.

**Antibiotics**	**Resistance gene marker**	**No. of strains (%) positive for antibiotic resistance genes**			**No. of strains showing MIC value of antibiotics (mg/L)**										
		**MRSE**	**MSSE**	**Total**	**0.125**	**0.25**	**0.5**	**1**	**2**	**4**	**8**	**16**	**32**	**64**	**>64**
Oxacillin	*mecA*	35 (67%)	0 (0%)	35 (67%)	0	**4**	**4**	**3**	0	0	**2**	0	**1**	0	**21**
Penicillin	*blaZ*	32 (62%)	18 (35%)	50 (96%)	0	0	0	**12**	0	**5**	**11**	**3**	**2**	**6**	**11**
Chloramphenicol	*catpC223*	2 (4%)	2 (4%)	4 (8%)	0	0	0	0	0	0	0	0	0	**2**	**2**
	*catpC221*	33 (63%)	14 (27%)	47 (90%)	0	0	0	0	0	5	7	***16***	**4**	**3**	**12**
	*catpC223 + catpC221*	2 (4%)	2 (4%)	4 (8%)	0	0	0	0	0	0	0	0	0	**2**	**2**
Erythromycin	*ermC*	25 (48%)	15 (28%)	40 (76.9%)	2	3	1	***4***	***3***	0	**1**	**1**	**3**	0	**22**
	*msrA*	14 (27%)	4 (8%)	18 (35%)	0	0	0	0	0	0	**3**	0	**3**	0	**12**
	*ermC + msrA*	13 (25%)	4 (8%)	17 (33%)	0	0	0	0	0	0	**3**	0	**3**	0	**11**
Clindamycin	*mphC*	16 (31%)	9 (17%)	25 (48%)	15	1	1	***5***	0	0	0	0	0	0	**3**
Gentamicin	*aac6′-aph2′*	32 (62%)	14 (27%)	47 (90%)	28	3	1	3	0	3	0	0	**4**	**1**	**4**
	*aph3′*	18 (35%)	7 (13%)	25 (48%)	6	5	0	1	1	4	0	0	**4**	**1**	**3**
	*aac6′-aph2′+ aph3′*	17 (33%)	5 (10%)	22 (42%)	6	4	0	1	1	3	0	0	**3**	**1**	**3**
Tetracycline	*tetK*	32 (62%)	15 (29%)	47 (98%)	0	0	0	4	6	0	***4***	**1**	**5**	**21**	**6**

*Resistant: Bold; Intermediate: Bold italic; Sensitive: Normal*.

**Table 2:** Results of MIC obtained with fluoroquinolones and mutations identified in *gyrA, gyrB, parC*, and *parE* among *S. epidermidis* strains isolated from the infected eye and healthy conjunctiva.

**Strain designation (no. of strains) from**		**MIC of fluoroquinolones (mg/L)**				**QRDR showing mutation in genes for**			
**Infected eye**	**Healthy Conjunctiva**	**MXF**	**GAT**	**OFX**	**CIP**	***gyrA***	***gyrB***	***parC***	***parE***
409, 1520, 1429 (3)	N69OD (1)	**4**	**4**	**32**	>**64**	S84 → F	NIL	S80 → F	NIL
–	N63OD, N95OS (2)	***1***	**2**	**8**	**4**				
1362 (1)	–	0.5	**2**	**16**	>**64**				
1497 (1)	–	>**64**	>**64**	>**64**	**32**				
1558 (1)	–	**2**	**16**	**16**	**32**				
1606 (1)	–	**2**	**32**	**2**	**2**				
–	N64OS (1)	**2**	**2**	**16**	**16**				
108, 1121, 1184, 1515 (4)	–	**4**	**4**	**32**	>**64**	S84 → F	NIL	S80 → F	NIL
								D84 → C^*^	
866, 1001, 1386 (3)	–	**4**	**4**	**32**	>**64**	S84 → Y	NIL	S80 → Y	NIL
–	N80OS (1)	>**64**	>**64**	>**64**	>**64**				
–	N89OS (1)	**2**	**2**	**8**	**4**				
1142W (1)	–	***1***	***1***	**2**	**32**	NIL	NIL	NIL	N404 → S
–	N78OD (1)	**2**	**4**	**8**	**4**				
1502 (1)	N79OS (1)	**4**	**8**	>**64**	>**64**	S84 → Y		S80 → F	
–	N3OD (1)	>**64**	>**64**	>**64**	>**64**	S84 → F	NIL	S80 → Y	NIL
–	N67OS (1)	**2**	**4**	**16**	**16**				
–	N74OS, N101OD (2)	**2**	**2**	**16**	**16**	S84 → F	NIL	NIL	NIL
–	N91OD, N93OSW (2)	0.25	0.25	***1***	***1***	NIL	NIL	D84 → A	NIL
1033 (1)	–	**2**	**1**	**32**	>**64**	S84 → F	NIL	S80 → F	NIL
								V82 → L^*^	
								D84 → C^*^	
1122 (1)	–	>**64**	>**64**	>**64**	**8**	S84 → F	NIL	NIL	N404 → S
1123 (1)	–	***1***	**2**	**16**	***1***	S84 → F	NIL	S80 → F	N404 → S
1056 (1)	–	**2**	**2**	>**64**	>**64**	S84 → F	NIL	D84 → Y	N404 → S
1150 (1)	–	**4**	**4**	**32**	**8**	S84 → F	NIL	D84 → Y	N404 → S
–	N96OD (1)	**32**	**32**	>**64**	**64**	S84 → Y	NIL	NIL	NIL
–	N98OD (1)	0.125	0.125	0.25	0.25	NIL	NIL	S80 → F	NIL
1163, 1528 (2)	N1OD, N5OD, N6OS, N16OD, N18OD, N41OD, N46OD, N47OD, N52OD, N53OS, N81OD, N85OD, N94OD (13)	0.125	0.125	0.5	0.25	NIL	NIL	NIL	NIL

*MXF, Moxifloxacin; GAT, Gatifloxacin; OFX, Ofloxacin; CIP, Ciprofloxacin. Resistant: Bold; Intermediate: Bold italic; Sensitive: Normal*.

* *Novel mutations found in this study*.

### Antibiotic resistance genes (ARG) and gyrase mutation

Majority of *S. epidermidis* isolates showing phenotypic resistance to multiple antibiotics carried ARGs (Table 1). Of the 36 fluoroquinolone resistant *S. epidermidis*, 32 strains comprising 20 from the infected eye and 12 from healthy conjunctiva showed mutations S84 → F/Y in *gyrA* gene, but none in the *gyrB* gene (Table 2). Similarly, 31 strains comprising 19 isolates from the infected eye and 12 from healthy conjunctiva showed a known mutation in *parC* gene. All isolates showing high MIC value for fluoroquionolones (especially that ≥ 64 mg/L) had mutation in either *gyrA* gene or *gyrA* and *parC* genes. In addition, five isolates from infected eye also showed novel mutation D84 → C and one to V82 → L in *parC*, not reported earlier. Of the six strains, five isolates from the infected eye and one from healthy conjunctiva showed mutation N404 → S in the *parE* gene (Table 2).

### Virulence profile of *S. epidermidis*

All the *S. epidermidis* strains harbored *icaA* and *icaD* genes. Of the 52 isolates, 20 from infected eye and 27 from healthy conjunctiva contained ACME operon (**Table 4**). Similarly 22 isolates from the infected eye and 28 from healthy conjunctiva amplified a fragment of *IS256* indicating the presence of insertion sequence in *S. epidermidis* (Table S4, **Figure 2**). These observations thus suggest that virulence determinants were present in *S. epidermidis* strains, irrespective of sources of isolation.

### SCC*mec* typing

**Figure 1:**
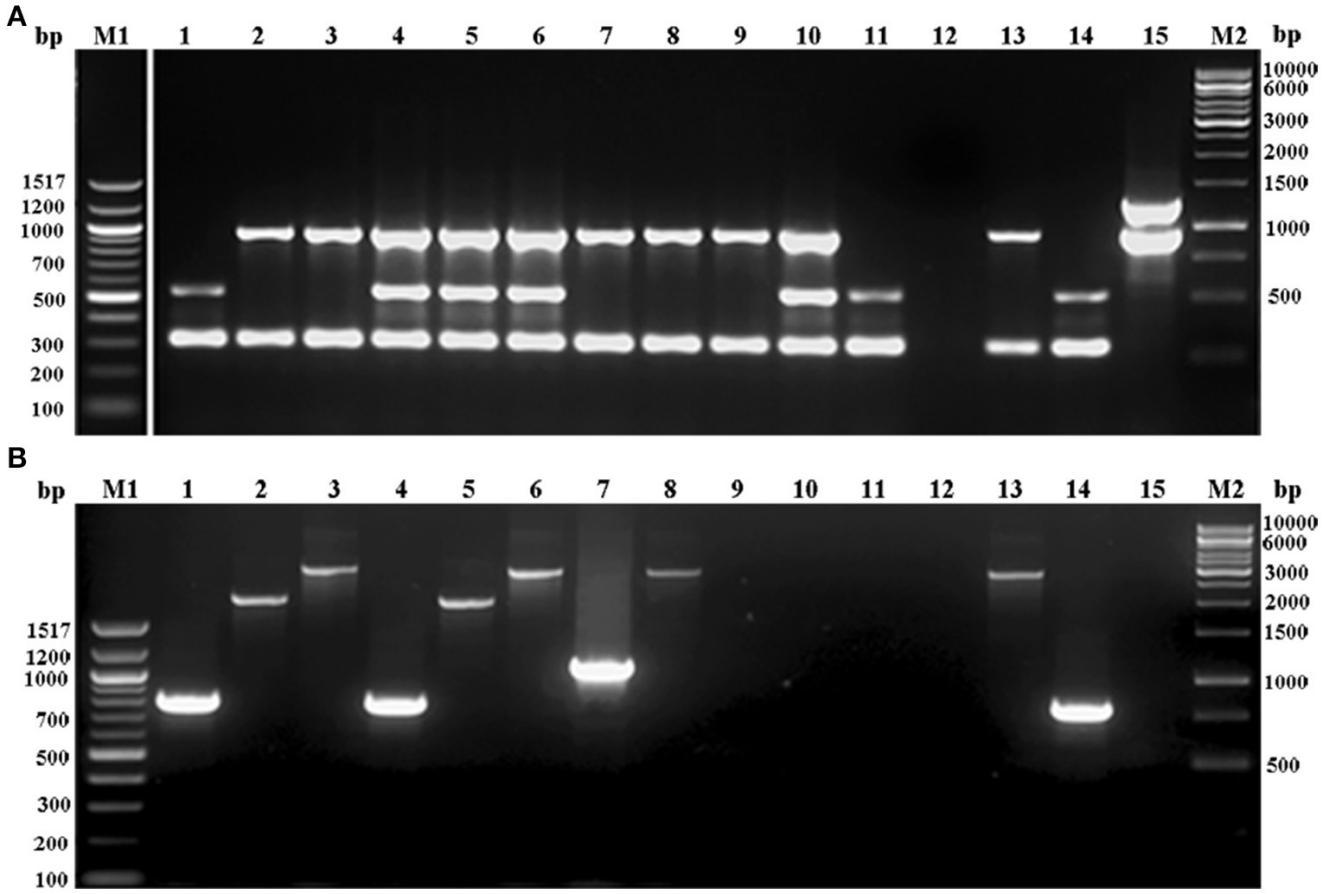
Agarose gel electrophoresis of multiplex PCR employed for **(A)** *ccr* complex typing (MPCR1), **(B)**. *mec* complex typing (MPCR2) of *S. epidermidis* strains. Lane M1: 100bp ladder; lane 1: *S. epidermidis* strain 866; lane 2: *S. epidermidis* strain 1056; lane 3: *S. epidermidis* strain N6OS; lane 4: *S. epidermidis* strain 1150; lane 5: *S. epidermidis* strain N91OD; lane 6: *S. epidermidis* strain 108; lane 7: *S. epidermidis* strain N96OD; lane 8: *S. epidermidis* strain 1558; lane 9: *S. epidermidis* strain N89OD; lane 10: *S. epidermidis* strain 1386; lane 11: *S. epidermidis* strain N81OD; lane 12: *S. epidermidis* strain 1163; lane 13: *S. aureus* strain Mu50; lane 14: *S. epidermidis* strain 1295; lane 15: *S. epidermidis* strain 12228; lane M2, 1kb ladder.

Of the MRSE strains, 25 belong to known SCC*mec* types of which 17 strains carried SCC*mec* type IV, four type V, two type II, and two carried UT4 (Table 3, Figure 1). The remaining 10 MRSE isolates showed four different combinations of *mec* complex and *ccr* complex, not reported earlier and thus do not fall within the traditional classification scheme. Of these strains, seven carried multiple *ccr* complexes and SCC mercury element (SCC*mer*). One strain from infected eye belonging to UT4 was also positive for SCC*mer* (Table 3, Figure 1). Although ten of the 17 MSSE isolates failed to amplify *mec* and *ccr* complex, five isolates carried *ccr*A2B2, and one isolate carried *ccr*C1 but lacked *mec* complex. One of the MSSE isolates carried more than one *ccr* type (A2B2, C1) and SCC*mer* (Table 3, Figure 1). Strains belonging to unreported SCC*mec* types (UT12 – UT14) showed a high level of oxacillin resistance (data not shown).

**Table 3:** Distribution of SCC*mec* types and SCC*mer* element among *S. epidermidis* strains isolated from the infected eye and healthy conjunctiva.

***SCCmec* type**	**No. of strains**	**No. of strains from**		***mecA* gene**	***ccr* complex**	***mec* complex**	**SCC*mer***
		**Infected eye**	**Healthy conjunctiva**				
V	4	1	3	+	C1	C2	0
IV	17	9	8	+	A2B2	B	0
II	2	0	2	+	A2B2	A	0
UT4	2	1^*^	1	+	A2B2, C1	C2	1^*^
UT11	2	0	2	+	A2B2, C1	B	2
UT12	5	4	1	+	A2B2, C1	A	5
UT13	2	0	2	+	A2B2	C2	0
UT14	1	1	0	+	A2B2	C2+	0
UNT-I	5	1	4	−	A2B2	−	0
UNT-II	1	1	0	−	A2B2, C1	−	1
UNT-III	1	0	1	−	C1	−	0
Absent	10	5	5	−	−	−	0

UT, Unnamed type; UNT, Untypeable;

* *One of the UT4 strain carried SCCmer*.

### Genomic diversity

**Figure 2:**
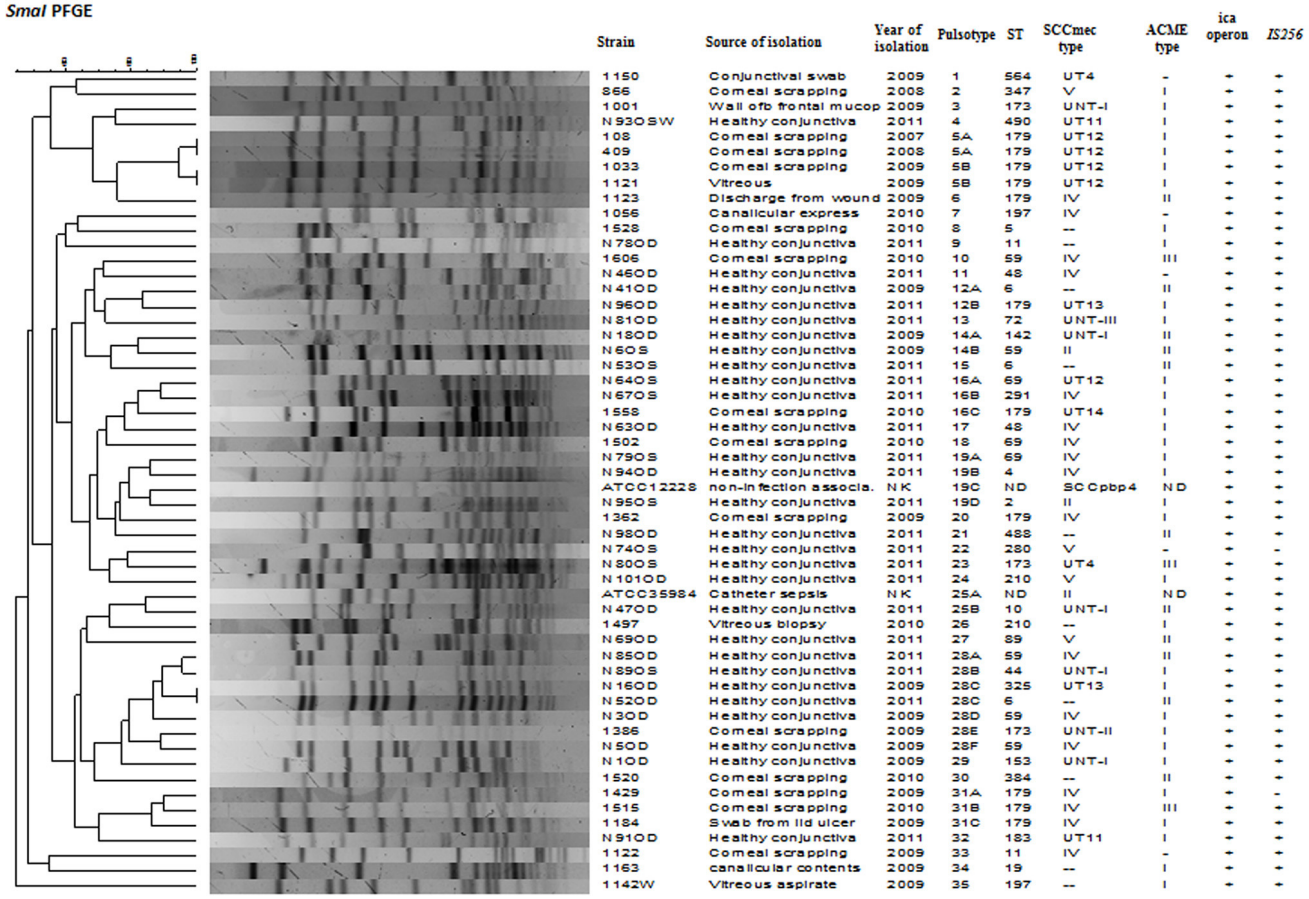
Dendrogram representation (Dice co-efficient) for macro-restriction banding patterns of *S. epidermidis* strains isolated from infected eye and healthy conjunctiva along with ATCC reference strains, generated by Pulsed-field gel electrophoresis of total chromosomal DNA digested with *SmaI* restriction enzyme. A correlation between Pulsotype, ST, SCC*mec* type, ACME, *ica* operon, *IS256*, and source of isolation is also shown. UT: Unnamed type, UNT: Untypeable, ND: not detected, NK: not known.

**Figure 3:**
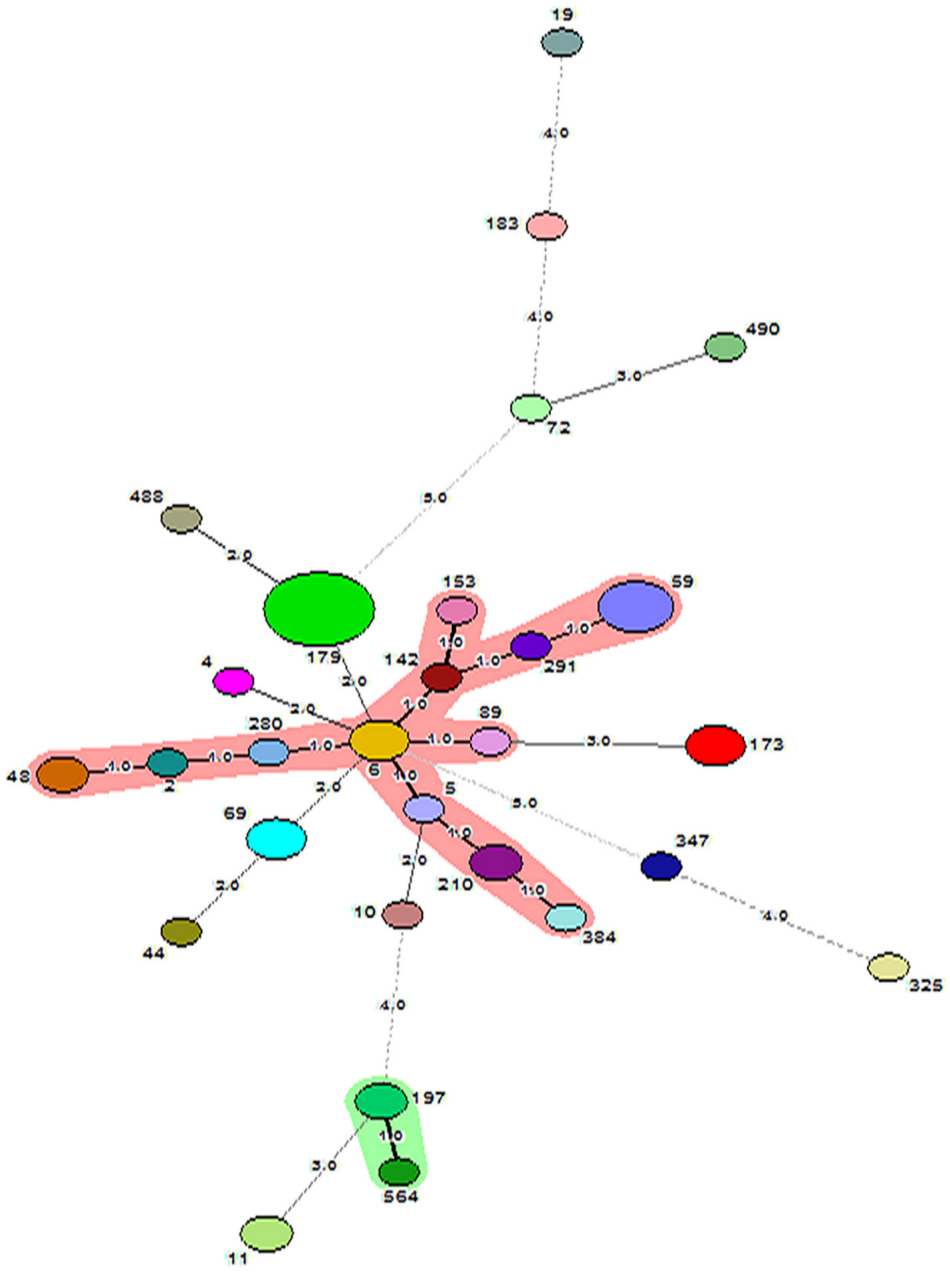
Minimum Spanning Tree showing relationship between different STs assigned by the analysis of MLST data. Each node represents one sequence type and the corresponding ST is given beside the node. The size of each node is directly proportional to the number of strains included under that ST. The number given on the strings connecting the nodes stands for the number of genes by which the strains under those STs differ from each other. Bold lines connect types that are identical for 6 loci, solid lines connecting types identical for ≥4 but ≤ 6 locus and dotted lines connecting STs differing from each other by ≥4 genes out of seven gene loci. CC59 and CC197 are represented as shaded area.

*SmaI* restriction digestion of DNA from *S. epidermidis* generated 14–21 bands, classifying the isolates into 35 pulsotypes, 16 subtypes, and three identical pairs (Table 4, Figure 2). Of the 52 *S. epidermidis* strains, 47 comprising 20 from infected eye and 27 from healthy conjunctiva harbored ACME operon of which 33 belonged to ACME type I, 11 to type II, and three to type III, respectively (Table 4). MLST analysis of the *S. epidermidis* strains identified 28 distinct STs of which ST179 was found in 43% of infected eye isolates followed by ST59 that was found in 13% of healthy conjunctiva isolates (Table 4). Partitioning analysis of the MST generated two CCs of which CC59 included 12 STs (ST59, ST6, ST210, ST48, ST291, ST142, ST153, ST89, ST5, ST384, ST280 and ST2) and CC197 includes two STs (ST197 and ST564). ST6 seems to be the founder of CC59 according to the eBURST algorithm. The remaining 14 STs were singletons of which ST179 comprised of 11 strains, ST69 and ST173 three strains, ST11 two strains, ST4, ST10, ST19, ST44, ST72, ST183, ST325, ST347, ST488, and ST490 one strain each (Table 4, Figure 3). A good correlation was found between high level resistance to fluoroquinolones and major sequence type, ST179.

**Table 4:** Distribution of ST, PFGE types, SCC*mec* types and virulence genes among *S. epidermidis* isolated from the infected eye and from healthy conjunctiva.

**ST (no. of strains, %)**	**PFGE types**	**SCC*mec* types (no. of strains)**	**No. of strains showing the presence of virulence factors**					
			**ACME I**	**ACME II**	**ACME III**	***icaA***	***icaD***	***IS256***
**INFECTED EYE**								
ST179 (10, 43.4%)	5	UT14 (1) UT12 (4) IV (5)	8	1	1	10	10	9
ST197 (2, 8.6%)	2	IV (1) Absent (1)	1	0	0	2	2	2
ST173 (2, 8.6%)	2	UNT-I (1) UNT-II (1)	2	0	0	2	2	2
Other STs (9, 39.1%)	9	V (1), IV (3), UNT-IV (1), Absent (4)	5	1	1	9	9	9
**HEALTHY CONJUNCTIVA**								
ST59 (4, 13.7%)	4	IV (3) II (1)	2	2	0	4	4	4
ST6 (3, 10.3%)	3	Absent (3)	0	3	0	3	3	3
ST48 (2, 6.8%)	2	IV (2)	1	0	0	2	2	2
ST69 (2, 6.8%)	2	IV (1) UT12 (1)	2	0	0	2	2	2
Other STs (18, 62%)	16	IV (2), V (3), II (1), UNT-I (4), UT11 (2), UT13 (2), UNT-IV (1), UNT-III (1), Absent (2)	12	4	1	18	18	17

*ST, sequence type; PFGE, pulsed field gel electrophoresis; MLST, multi-locus sequence typing; UT, Unnamed type; UNT, Untypeable*.

### SID for MLST, SCC*mec*, and PFGE

We calculated SID for all the three typing techniques. The values derived for MLST, SCC*mec* typing and PFGE were 0.95, 0.85, and 0.97, suggesting the existence of genomic diversity among the isolates irrespective of sources of isolation. Overall result suggests that PFGE and MLST were more discriminatory than SCC*mec* typing to study genomic diversity among *S. epidermidis*.

### Statistical analysis

**Figure 4:**
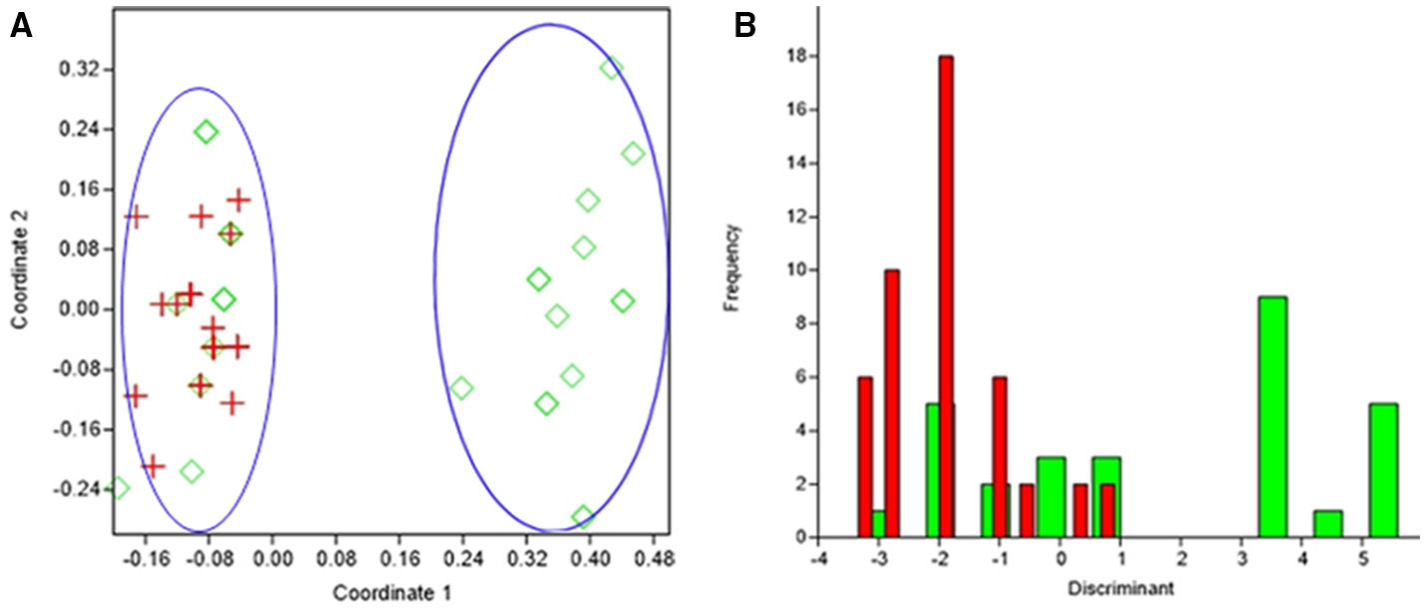
Results obtained by statistical analysis using phenotypic and genotypic data. **(A)** Principal Coordinates Analysis (PCoA) of *S. epidermidis* from infected eye, (IE, red), and isolates from healthy conjunctiva (HC, green). **(B)** Discriminant analysis of the isolates of *S. epidermidis* from infected eye, (IE, red), and isolates from healthy conjunctiva (HC, green). Negative values belong to IE isolates and positive value to HC isolates.

PCoA can segregate isolates belonging to the healthy conjunctiva and infected eye, except for few isolates from healthy conjunctiva (axis one was used for the highest percentage of representation), with 33% of explained variance (Figure 4A). From DA graph, it was clear that, whereas isolates from infected eye grouped within more negative values, majority isolates from healthy conjunctiva grouped within positive values (Figure 4B). Predominant biomarkers were determined by calculating the coefficient of discriminant function and considered when the value was equal to 0.5 or >0.5. Whereas infected eye isolates are discriminating in the biomarker of resistance to gatifloxacin (−3.332), ofloxacin (−3.332), and tetracycline (−2.131), the healthy conjunctiva isolates were discriminating in resistance to moxifloxacin (0.64542), ciprofloxacin (0.69144), and erythromycin (1.5531).

## Discussion

*S. epidermidis* has been reported causing indwelling medical device infections, such as prosthetic valve endocarditis, and intra-cardiac abscesses leading to high mortality (Otto, 2009). The incidence of *S. epidermidis* in ocular disease is more frequent than any other species including *S. aureus* (Duggirala et al., 2007; Flores-Páez et al., 2015). The frequency of methicillin resistance among disease-associated isolates was higher than commensal strains (Duggirala et al., 2007). Several studies have shown that MDR is more frequent in MRSE than MSSE (Cherifi et al., 2013, 2014; Deplano et al., 2016). Also, strains from the infected eye were more resistant to non-beta-lactam antibiotics than those from the healthy conjunctiva (Duggirala et al., 2007; Cherifi et al., 2013, 2014). This study also showed that *S. epidermidis* isolates are multidrug resistant, irrespective of methicillin-resistance and sources of isolation. Similar to other workers 70% of isolates comprising isolates from healthy conjunctivae and the infected eye carried *catpC221* or *catpC223* or both gene(s) encoding for chloramphenicol resistance (Cherifi et al., 2013; Bispo et al., 2014). A good correlation was found between the presence of *catp223* and/or *catp221* gene(s) and chloramphenicol resistance. The *catpC221* gene encoding for the resistance to chloramphenicol was reported previously in CoNS, especially in *S. pseudintermedius* of veterinary origin (Perreten et al., 2010; Kern and Perreten, 2013). A small percentage of *S. epidermidis* showed resistance to clindamycin and gentamicin a finding similar to those workers who reported resistance to these antibiotics among isolates from ocular and blood stream infections (Kern and Perreten, 2013; Bispo et al., 2014). In contrast, a high percentage of MLS_B_ resistance gene *ermC* encoding for macrolide-lincosamide-streptograminB resistance was present among *S. epidermidis* isolates comprising 12 from the infected eye and 24 from the healthy conjunctiva (Argudin et al., 2011). Interestingly, most frequent ARG found in this study was *tetK*, though many of the isolates carrying this gene were phenotypically sensitive to tetracycline. Like Argudin et al. (2011) the bifunctional aminoglycoside resistance-conferring gene *aac6*′*-aph2*″ was present in majority strains of which only 34% were phenotypically resistant to gentamicin. The commensal isolates were reported showing multidrug resistance (Li et al., 2009). Also, in this study, isolates from the healthy conjunctiva showed resistance to chloramphenicol, fluoroquinolones, erythromycin and tetracycline. These observations thus suggest that even the commensal strains can acquire resistance without being exposed to the antibiotics, therefore, acts as reservoirs for ARGs.

Fluoroquinolones are the preferred antibiotics for the treatment of staphylococcal ocular infections. Use of inappropriate dosage and overexposure of the bacteria to the antibiotic had facilitated the emergence of resistance to this drug. Mutation(s) in the *gyrA, gyrB, parC*, and *parE* genes is the primary mechanism of fluoroquinolone resistance. Like other workers, we also did not find any mutation in *gyrB* among *S. epidermidis* (Yamada et al., 2008). These workers have shown mutations in QRDR of *gyrA, gyrB, parC*, and *parE* genes in ~50% of *S. epidermidis* isolates from the human conjunctiva. However, the results of the present study showed an increase in mutation in QRDR up to 70% until 2011. We like other workers also found a mutation in *gyrA* (Ser84 → F/Y) in *S*. *epidermidis* strains that showed a significant association with high level of resistance to fluoroquinolones (Yamada et al., 2008; Betanzos-Cabrera et al., 2009; Bispo et al., 2013). In addition to the reported mutation in *parC* (Ser80 → F/Y and D → A), we found two novel mutations (D84 → C and V82 → L). These mutations do not alter the level of MIC significantly for any of the tested fluoroquinolones. Although mutation in *gyrA* gene confers resistance to fluoroquinolone, mutations in *parC* or *parE* do not alter the level of fluoroquinolones resistance. We found high MIC value for ciprofloxacin (32 mg/L) in one of the strain 1142W that showed mutation only in *parE* gene but its role in high level of quninolone resistance is not known (Yamada et al., 2008). We also did not find any association of high level of MIC to ciprofloxacin in a strain that had mutation in only *parE* gene. It is likely that other mechanism such as efflux of quinolone may be responsible for quinolone resistance leading to high level of ciprofloxacin resistance in this strain (Betanzos-Cabrera et al., 2009).

Several studies showed that *ica* operon is more prevalent among *S*. *epidermidis* isolated from a variety of infections than the commensals (Cherifi et al., 2013; Du et al., 2013). In contrast, all isolates of the present study carried *ica* operon irrespective of sources of isolation. These findings are similar to those workers who also reported the presence of *ica* operon among the isolates from both infected eye and healthy conjunctiva (Duggirala et al., 2007; Cherifi et al., 2013; Flores-Páez et al., 2015). Thus, this study support the proposal of eliminating detection of *ica* gene as a factor for differentiating commensal from invasive isolates (Wisplinghoff et al., 2003). Similarly, ACME operon was present in 47 *S*. *epidermidis* strains. This finding is in contrast to those workers who reported less number of *S. epidermidis* showing the presence of ACME operon (Cherifi et al., 2013; Du et al., 2013; Deplano et al., 2016). These authors also demonstrated that ACME was found more commonly in commensals than those isolated from infections (Du et al., 2013). In contrast, we did not find much difference in the distribution of ACME operon among *S. epidermidis* with regard to sources of isolation.

The majority of isolates from the infected eye and healthy conjunctiva were methicillin-resistant and carried the *mecA* gene. We did not find the presence of *mecC* (a variant of *mecA*) in any of the *S. epidermidis* isolates. These findings thus suggest that there is no significant difference of oxacillin resistance and the presence of *mecA* gene between sources of isolation. MRSE isolates were mostly multidrug resistant and belong to SCC*mec* type IV. These findings are similar to those workers who also reported the presence of *SCCmec* type IV in *S. epidermidis* (Wisplinghoff et al., 2003; Duggirala et al., 2007; Cherifi et al., 2014). The findings of this study thus suggest that there is mobility of SCC*mec* cassette among *S. epidermidis*, similar to that reported in *S. aureus* (Miragaia et al., 2007). Also, nine *S. epidermidis* isolates comprising six from the infected eye and three from healthy conjunctiva showed amplification of mercury resistance and J-region suggesting the presence of *SCCmercury* operon. To the best of our knowledge, this is the first report of the presence of SCC*mercury* among ocular isolates of *S. epidermidis*, although this element was reported earlier in *S. aureus* strains (Kondo et al., 2007).

A novel combination of ccr A2B2 and mecC2, designated as UT13, was present among *S. epidermidis* strains and was not included in the earlier classification scheme (Zong et al., 2011). We also found amplification of more than one *ccr* complex among few isolates designated as UT11 and UT12 indicating the presence of composite SCC*mec* types. Although the presence of more than one type of *ccr* complex was reported earlier in *S. epidermidis*, 17 MSSE isolates from this study were untypeable because none of the strains amplified either one or both of the complexes (Kondo et al., 2007; Cherifi et al., 2014). Also, we did not find the presence of Ψ*SCCmec* complex designated as a pseudo-SCC*mec* element, carrying *mec* complex but lacking *ccr* complex, in any of the strains. The heterogeneity of isolates including those from healthy conjunctiva indicates that *S. epidermidis* can act as a reservoir and play a role in the emergence of new types of SCC*mec* elements. It can happen due to acquisition or loss of SCC*mec* element which is frequent in CoNS (Zong et al., 2011).

MLST analysis revealed that ST179 is the most common ST found in 43% of infected eye isolates of *S. epidermidis*. This ST179 was reported earlier in single isolate each from an ocular strain in Brazil in 2009 and Iran in 2016 (Cherifi et al., 2013; Bispo et al., 2014). The second most frequent ST was ST59 and found in one strains from infected eye and three from healthy conjunctiva. This ST59 along with other STs was reported in strains isolated from blood culture, ocular infection and the healthy conjunctiva and suggested their association with the eye disease (Mertens and Ghebremedhin, 2013; Bispo et al., 2014; Soroush et al., 2016). There is good correlation between types of STs, ST179, and ST59 with source of isolation. These findings are in contrast to those workers who reported that lineage ST2 is most frequent in ocular infection and lineage ST5 was abundant in healthy conjunctiva isolates showing a substantial difference between isolates from ocular disease and healthy conjunctiva (Flores-Páez et al., 2015). However, a study conducted on clinical isolates in US hospital on bone and joint infection showed association of ST5 lineage with disease (Mendes et al., 2012; Cremniter et al., 2013). High level of fluoroquinolone resistance was significantly associated with sequence type ST179 of *S. epidermidis*. This observation is similar to those workers who also reported association of major clones of HA-MRSA with high-level resistance to fluoroquinolones (Horváth et al., 2012; Knight et al., 2012; Holden et al., 2013).

PFGE is the most appreciated method used for short-term epidemiological investigation. In this study, PFGE revealed that isolates from both infected eye and the healthy conjunctiva were genetically diverse. It can be seen that out of 35 pulsotypes; the infected eye isolates showed 16 pulsotypes, and the healthy conjunctiva isolates showed 17 pulsotypes. Only two pulsotypes was common among members of both the groups. Several studies also reported diversity among *S. epidermidis* (Cherifi et al., 2013; Martínez-Meléndez et al., 2016). Though strains from the infected eye and healthy conjunctiva clustered separately, there was high diversity within the members of the same group. This finding is in agreement with those workers who reported polyclonality among *S. epidermidis* isolated from the same host (Ueta et al., 2007).

It was clear from statistical analysis, that there is no specific discriminant marker to differentiate isolates of eye infection from healthy conjunctiva. However, the resistance to fluoroquinolones, erythromycin, and tetracycline can discriminate infected eye strains from the healthy conjunctiva isolates. This study thus suggests that few healthy conjunctiva isolates had characteristics of infected eye isolates.

We conclude that *S. epidermidis* strains are multidrug resistant, virulent and carried *mecA* gene, irrespective of sources of isolation. Although majority strains carried known types of SCC*mec*, seven strains carried unreported SCC*mec* types including SCC*mercury*. To the best of our knowledge, this is the first report of the presence of SCC*mercury* among ocular isolates of *S. epidermidis*. Few strains besides possessing known mutation in *gyrA, parC*, and *parE*, had additional mutation in *parC* gene. *IS256* cannot be used as a marker to differentiate isolates from infected eye and healthy conjunctiva. Statistical analysis suggests that few healthy conjunctiva isolates had characteristics similar to infected eye isolates. A noteworthy finding was the presence of ST179 in 43% of infected eye isolates an observation rarely reported among *S. epidermidis*. PFGE and MLST analysis indicates genomic diversity among them irrespective of sources of isolation.

## Author contributions

SJ, SP, and DVS designed the research; SJ and SP participated in most of the experiment; SJ, and SP analyzed the data; KN performed the statistical analysis; SJ drafted the manuscript; SJ, SP, KN, and DVS revised the manuscript.

### Conflict of interest statement

The authors declare that the research was conducted in the absence of any commercial or financial relationships that could be construed as a potential conflict of interest.
