# Supplementary material for: Identification of Major Sequence Types among Multidrug-Resistant Staphylococcus epidermidis Strains Isolated from Infected Eyes and Healthy Conjunctiva
Source: Front Microbiol. 2017 Aug 4;8:1430. doi: 10.3389/fmicb.2017.01430 (PMC5543311; doi:10.3389/fmicb.2017.01430)
Supplement: Supplementary file 1 [file Table1.PDF]

Supplimentary material

Table S1. Phenotypic and genotypic data obtained with *S. epidermidis* isolated from infected eye.

Strain	Infection type	Pulsotype	ST	SCC*mec* type	*mecA*	ACME type	*icaA*	*icaD*	*IS256*	OXA	CHL	VAN	CFZ	MXF	GAT	OFX	CIP	ERY	GEN	CLI	TET
108	K	5A	179	UT12	+	I	+	+	+	**R**	**R**	S	S	**R**	**R**	**R**	**R**	S	S	S	**R**
409	K	5A	179	UT12	+	I	+	+	+	**R**	S	S	S	**R**	**R**	**R**	**R**	S	S	S	**R**
866	K	2	347	V	+	I	+	+	+	**R**	S	S	S	**R**	**R**	**R**	**R**	**R**	**R**	S	**R**
1001	OTHERS	3	173	UNT-I	-	I	+	+	+	S	**R**	S	S	**R**	**R**	**R**	**R**	**R**	S	S	**R**
1033	K	5B	179	UNT-II	+	I	+	+	+	**R**	S	S	S	**R**	**R**	**R**	**R**	**R**	S	S	**R**
1056	C	7	197	IV	+	-	+	+	+	**R**	**R**	S	S	**R**	**R**	**R**	**R**	**R**	**R**	S	S
1121	E	5B	179	UT12	+	I	+	+	+	**R**	S	S	S	**R**	**R**	**R**	**R**	S	S	S	**R**
1122	K	33	11	IV	+	-	+	+	+	**R**	**R**	S	S	**R**	**R**	**R**	**R**	**R**	S	S	S
1123	OTHERS	6	179	IV	+	II	+	+	+	**R**	S	S	S	**R**	**R**	**R**	S	S	S	S	**R**
1142W	OTHERS	35	197	NA	-	I	+	+	+	S	**R**	S	S	**R**	**R**	**R**	**R**	**R**	S	S	**R**
1150	C	1	564	UNT-IV	+	-	+	+	+	**R**	**R**	S	S	**R**	**R**	**R**	**R**	**R**	**R**	S	**R**
1163	OTHERS	34	19	NA	-	I	+	+	+	S	**R**	S	S	**R**	**R**	**R**	**R**	S	**R**	S	**R**
1184	OTHERS	31C	179	IV	+	I	+	+	+	**R**	**R**	S	S	**R**	**R**	**R**	**R**	S	**R**	S	**R**
1362	K	20	179	IV	+	I	+	+	+	**R**	**R**	S	S	S	**R**	**R**	**R**	S	**R**	S	**R**
1386	K	28E	173	UNT-II	-	I	+	+	+	S	**R**	S	S	**R**	**R**	**R**	**R**	**R**	S	S	**R**
1429	K	31A	179	IV	+	I	+	+	-	**R**	S	S	S	**R**	**R**	**R**	**R**	S	S	S	**R**
1497	E	26	210	NA	-	I	+	+	+	S	**R**	S	S	**R**	**R**	**R**	**R**	**R**	S	**R**	**R**
1502	K	18	69	IV	+	I	+	+	+	**R**	**R**	S	S	**R**	**R**	**R**	**R**	**R**	S	**R**	**R**
1515	OTHERS	31B	179	IV	+	III	+	+	+	**R**	**R**	S	S	**R**	**R**	**R**	**R**	**R**	S	**R**	**R**
1520	K	30	384	NA	-	II	+	+	+	S	**R**	S	S	**R**	**R**	**R**	**R**	**R**	S	**R**	**R**
1528	K	8	5	NA	-	I	+	+	+	S	**R**	S	S	**R**	**R**	**R**	**R**	**R**	S	**R**	**R**
1558	K	16C	179	UT14	+	I	+	+	+	**R**	**R**	S	S	**R**	**R**	**R**	**R**	**R**	S	**R**	**R**
1606	K	10	59	IV	+	III	+	+	+	**R**	**R**	S	S	**R**	**R**	**R**	**R**	**R**	**R**	S	**R**

K=Keratitis; C= Conjunctivitis; E= Endocarditis; others= endophthalmitis, marsupialization of cyst, chronic dacryocystitis, traumatic cataract, canaliculitis, blepharitis and graft infiltrate. OXA= Oxacillin; CHL= Chloramphenicol; VAN= Vancomycin; CFZ= Cefazolin; MXF= Moxifloxacin; GAT= Gatifloxacin; OFX= Ofloxacin; CIP= Ciprofloxacin; ERY= Erythromycin; GEN= Gentamycin; CLI= Clindamycin; TET= Tetracycline. UT: Unnamed type, UNT: Untypeable; UT11 = A2B2 + C, B; UT12= A2B2 + C, A; UT13= A2B2, C; UT14= A2B2, C+; UNT-I = A2B2; UNT-II = A2B2 + C; UNT-III = C.

Table S2. Phenotypic and genotypic data obtained with *S. epidermidis* isolated from healthy conjunctiva.

Strain(s)	Pulsotype	ST	SCC*mec* type	*mecA*	ACME type	*icaA*	*icaD*	*IS256*	OXA	CHL	VAN	CFZ	MXF	GAT	OFX	CIP	ERY	GEN	CLI	TET
N1OD	9	153	UNT-I	-	I	+	+	+	S	**R**	S	S	**R**	**R**	**R**	**R**	**R**	**R**	**R**	**R**
N3OD	8D	59	IV	+	I	+	+	+	**R**	**R**	S	S	**R**	**R**	**R**	**R**	**R**	S	S	**R**
N5OD	28F	59	IV	+	I	+	+	+	**R**	**R**	S	S	S	S	S	S	**R**	**R**	S	**R**
N6OD	14B	59	II	+	II	+	+	+	**R**	**R**	S	S	**R**	S	S	S	**R**	**R**	S	**R**
N16OD	28C	325	UT13	+	I	+	+	+	**R**	**R**	S	S	S	S	S	S	**R**	**R**	S	**R**
N18OD	14A	142	UNT-I	-	II	+	+	+	S	**R**	S	S	S	S	S	S	**R**	**R**	S	**R**
N41OD	12A	6	NA	-	II	+	+	+	S	**R**	S	S	S	S	S	S	**R**	**R**	S	**R**
N46OD	11	48	IV	+	-	+	+	+	**R**	**R**	S	S	S	S	S	S	**R**	S	S	**R**
N47OD	25B	10	UNT-I	-	II	+	+	+	S	**R**	S	S	S	S	S	S	**R**	S	S	S
N52OD	28C	6	NA	-	II	+	+	+	S	**R**	S	S	S	S	S	S	**R**	S	S	S
N53OD	15	6	NA	-	II	+	+	+	S	**R**	S	S	S	S	S	S	**R**	S	S	**R**
N63OD	17	48	IV	+	I	+	+	+	**R**	**R**	S	S	**R**	**R**	**R**	**R**	**R**	S	S	**R**
N64OD	16A	69	UT12	+	I	+	+	+	**R**	**R**	S	S	**R**	**R**	**R**	**R**	**R**	S	S	**R**
N67OD	16B	291	IV	+	I	+	+	+	**R**	**R**	S	S	**R**	**R**	**R**	**R**	**R**	S	S	S
N69OD	27	89	V	+	II	+	+	+	**R**	**R**	S	S	**R**	**R**	**R**	**R**	**R**	**R**	S	**R**
N74OD	22	280	V	+	-	+	+	-	**R**	S	S	S	**R**	**R**	**R**	**R**	**R**	**R**	S	**R**
N78OD	9	11	NA	-	I	+	+	+	S	**R**	S	S	**R**	**R**	**R**	**R**	S	**R**	**R**	**R**
N79OS	19A	69	IV	+	I	+	+	+	**R**	**R**	S	S	**R**	**R**	**R**	**R**	**R**	S	S	S
N80OD	23	173	UT14	+	III	+	+	+	**R**	**R**	S	S	**R**	**R**	**R**	**R**	**R**	S	S	S
N81OD	13	72	UNT-III	-	I	+	+	+	S	S	S	S	S	S	S	S	**R**	S	S	**R**
N85OD	28A	59	IV	+	II	+	+	+	**R**	**R**	S	S	S	S	S	S	**R**	S	S	**R**
N89OD	28B	44	UNT-I	-	I	+	+	+	S	**R**	S	S	**R**	**R**	**R**	**R**	**R**	S	**R**	**R**
N91OD	32	183	UT11	+	I	+	+	+	**R**	**R**	S	S	S	S	S	S	**R**	S	**R**	**R**
N93OSW	4	490	UT11	+	I	+	+	+	**R**	S	S	S	S	S	S	S	**R**	S	**R**	S
N94OS	19B	4	IV	+	I	+	+	+	**R**	**R**	S	S	S	S	S	S	**R**	S	S	S
N95OS	19D	2	II	+	I	+	+	+	**R**	S	S	S	**R**	**R**	**R**	**R**	**R**	S	S	S
N96OS	12B	179	UT13	+	I	+	+	+	**R**	S	S	S	**R**	**R**	**R**	**R**	**R**	S	S	S
N98OS	21	488	NA	-	II	+	+	+	S	S	S	S	S	S	S	S	**R**	S	S	S
N101OS	24	210	V	+	I	+	+	+	**R**	S	S	S	**R**	**R**	**R**	**R**	**R**	S	S	S

OXA= Oxacillin; CHL= Chloramphenicol; VAN= Vancomycin; CFZ= Cefazolin; MXF= Moxifloxacin; GAT= Gatifloxacin; OFX= Ofloxacin; CIP= Ciprofloxacin; ERY= Erythromycin; GEN= Gentamycin; CLI= Clindamycin; TET= Tetracycline. UT: Unnamed type, UNT: Untypeable.; UT11 = A2B2 + C, B; UT12= A2B2 + C, A; UT13= A2B2, C; UT14= A2B2, C+; UNT-I = A2B2; UNT-II = A2B2 + C; UNT-III = C.

Table S3. Antibiotic resistance pattern obtained with *S. epidermidis* strains isolated from the infected eye and healthy conjunctiva.

Antibiotic resistance Pattern (Pattern type)	No. of isolates from				
	Infected eye		Healthy conjunctiva		
	MRSE	MSSE	MRSE	MSSE	Total
CHL, CIP, GAT, MXF, OFX, OXA, TET **(1)**	1	0	0	0	1
CIP, GAT, MXF,OFX, OXA, TET **(2)**	2	0	0	0	2
CIP, ERY, GAT, GEN, MXF, OFX, OXA, TET **(3)**	1	0	1	0	2
CHL, CIP, ERY, GAT, MXF, OFX, TET **(4)**	0	2	0	0	2
CIP, ERY, GAT, MXF, OFX, OXA, TET **(5)**	1	0	0	0	1
CHL, CIP, ERY, GAT, GEN MXF, OXA, OFX, **(6)**	1	0	0	0	1
CHL, CIP, ERY, GAT, MXF, OFX, OXA, **(7)**	1	0	3	0	4
GAT, MXF, OFX, OXA, TET **(8)**	1	0	0	0	1
CHL, CIP, ERY, GAT, GEN, MXF, OFX, OXA, TET **(9)**	2	0	1	0	3
CHL,MXF, GAT, OFX, CIP, GEN, TET **(10)**	0	1	0	0	1
CHL, CIP, GAT, GEN, MXF, OFX, OXA, TET **(11)**	1	0	0	0	1
CHL, CIP, GAT, GEN, OFX, OXA, TET **(12)**	1	0	0	0	1
CHL, CIP,ERY, GAT, MXF, OFX, TET **(13)**	0	1	0	0	1
CIP, GAT, MXF, OFX, OXA, TET **(14)**	1	0	0	0	1
CHL, CIP, CLI, ERY, GAT, MXF, OFX, TET **(15)**	0	3	0	1	4
CHL, CIP, CLI, ERY, GAT, MXF, OFX, OXA, TET **(16)**	3	0	0	0	3
CHL, CIP, CLI, ERY, GAT, GEN, MXF, OFX, TET **(17)**	0	0	0	1	1
CHL, CIP, ERY, GAT, MXF, OFX, OXA, TET **(18)**	0	0	3	0	3
CHL, ERY, GEN, OXA, TET **(19)**	0	0	2	0	2
CHL, ERY, GEN, MXF, OXA, TET **(20)**	0	0	1	0	1
CHL, ERY, GEN, TET **(21)**	0	0	0	2	2
CHL, ERY, OXA, TET **(22)**	0	0	2	0	2
CHL, ERY **(23)**	0	0	0	2	2
CHL, ERY, TET **(24)**	0	0	0	1	1
CHL, CIP, CLI, GAT, GEN, MXF, OFX, TET **(25)**	0	0	0	1	1
ERY, TET **(26)**	0	0	0	1	1
CHL, CLI, ERY, OXA, TET **(27)**	0	0	1	0	1
CLI, ERY, OXA **(28)**	0	0	1	0	1
CHL, CIP, OXA **(29)**	0	0	1	0	1
CIP, ERY, GAT, MXF, OFX, OXA, **(30)**	0	0	2	0	2
ERY **(31)**	0	0	0	1	1
CIP, ERY, GAT, MXF, OFX, OXA, **(32)**	0	0	1	0	1

CHL: Chloramphenicol; CIP: Ciprofloxacin; CLI: Clindamycin; ERY: Erythromycin; GAT: Gatifloxacin; GEN: Gentamycin; MXF: Moxifloxacin; OFX: Ofloxacin; OXA: Oxacillin; TET: Tetracycline

Table S4. Presence of virulence genes among *S.epidermidis* isolated from the infected eye and healthy conjunctiva.

Source and virulence factors	Presence of genes encoding for	No. of strains (%)		
		MRSE	MSSE	Total
Infected eye				
ica operon	*icaA*	16 (100)	7 (100)	23 (100)
	icaD	16 (100)	7 (100)	23 (100)
ACME operon	*arcA*	11 (68.7)	7(100)	18 (78.3)
	opp3AB	13 (81.2)	5 (71.4)	18 (78.3)
IS256 element	*IS256*	15 (93.7)	7 (100)	22 (95.7)
Healthy conjunctiva				
ica operon	*icaA*	19 (100)	10 (100)	29 (100)
	icaD	19 (100)	10 (100)	29 (100)
ACME operon	*arcA*	16 (84.2)	10 (100)	26 (89.6)
	opp3AB	15 (78.9)	4 (40)	19 (65.5)
IS256 element	*IS256*	18 (94.7)	10 (100)	28 (96.6)

MRSE: Methicillin Resistant *Staphylococcus epidermidis*; MSSE: Methicillin Susceptible *Staphylococcus epidermidis.* Ica: Intercellular adhesion; ACME: arginine catabolic mobile element; IS256: Insertion Sequence 256
